# Neuroendocrine Carcinoma of the Colon: Emergency Presentation of a Rare Disease With Poor Biology

**DOI:** 10.7759/cureus.19582

**Published:** 2021-11-14

**Authors:** Shakeel Masood, Ravi Gupta, Ashish Jaiswal, Gaurav Bhardwaj, Utkarsh Srivastav

**Affiliations:** 1 Surgical Gastroenterology, Ram Manohar Lohia Institute of Medical Sciences, Lucknow, IND; 2 General Surgery, All India Institute of Medical Sciences, Gorakhpur, IND; 3 Surgical Gastroenterology, Amrita Institute of Medical Sciences, Kochi, IND

**Keywords:** poor biology, neuroendocrine carcinoma colon, emergency presentation, colonic net, rare colonic tumor

## Abstract

Neuroendocrine tumors (NETs) are rare entities. Most common among them are gastroenteropancreatic neuroendocrine tumors (GEP-NETs) and pulmonary NETs. Most of them are indolent in nature. Colonic NETs are rare among GEP-NETs and mostly present with large size and with metastasis. Emergency presentation with hematochezia is rare in colonic NETs. This case report discusses a rare emergency presentation of colonic NETs and highlights their poor biological nature.

## Introduction

Neuroendocrine tumors (NETs) are generally rare and associated with low virulent behavior [1,2]. Among NETs, colon neuroendocrine carcinoma (NEC) accounts for 1% of all colorectal cancers (CRCs) [3]. Colon NECs are usually non-functional and present with vague abdominal pain, anorexia, unintentional weight loss, abdominal lump, and obstructive symptoms [4]. Hematochezia is a rare presentation among colonic NECs because most of these tumors are exophytic rather than ulcerative, but a similar presentation is common in rectal NECs [5]. Colonic NECs have a high mitotic index, leading to diseases of poor biological nature, and given the delayed nonspecific presentation and the lack of a well-established adjuvant therapy regimen, they are associated with poor overall survival [6]. In this report, we discuss a case of NEC of the colon with a rare emergency presentation and a poor response to adjuvant therapy leading to poor outcomes.

## Case presentation

**Figure 1:**
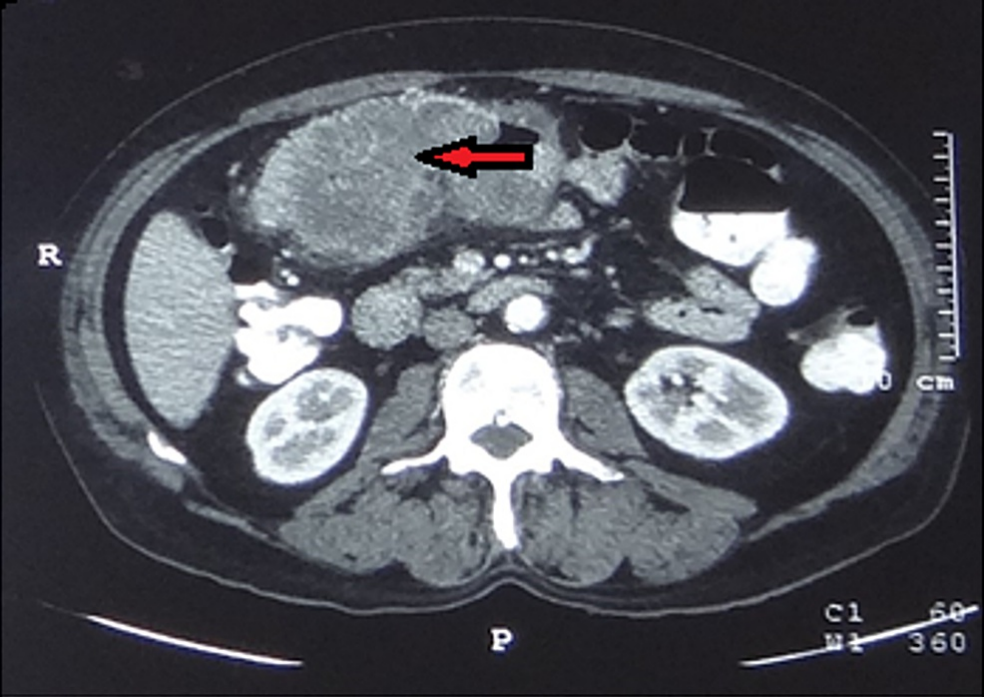
Preoperative CECT abdomen showing enhancing heterogenous transverse colon mass (arrow) CECT: contrast-enhanced computed tomography

A 65-year-old male with good performance status (Eastern Cooperative Oncology Group Scale 1) presented to the emergency department with episodes of haematochezia for two days. At the time of presentation, he had pallor and his vitals were within normal range except for tachycardia of 110 bpm. On abdominal examination, there was a vague lump in the left upper quadrant. On digital rectal examination, no growth was felt and the finger was stained with blood. The patient had already undergone a contrast-enhanced CT abdomen and thorax at the primary healthcare center, which was suggestive of transverse colon mass without any evidence of distant metastasis (Figure 1).

**Figure 2:**
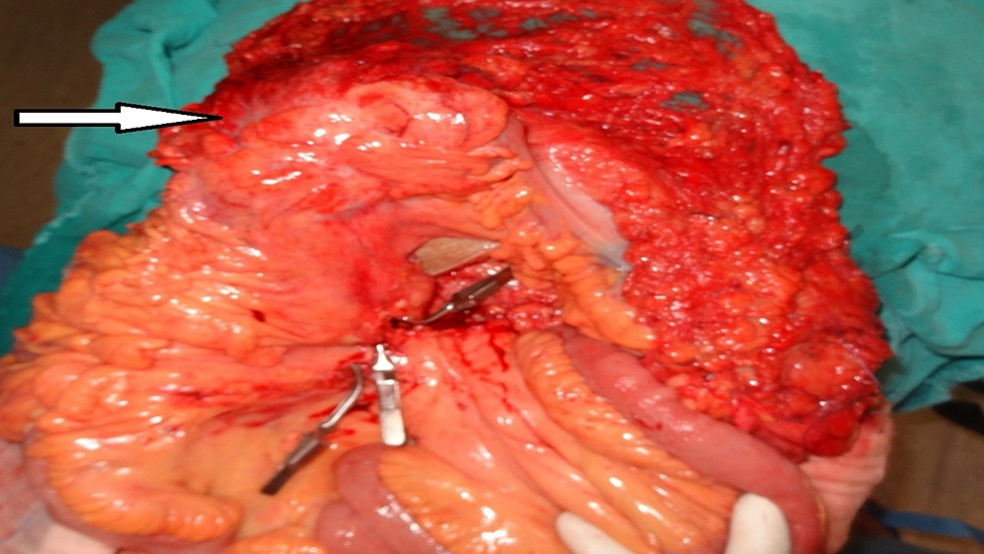
Transverse colon mass (arrow) and complete mesocolic excision (CME) with central vascular ligation (CVL) D3 extended right hemicolectomy

**Figure 3:**
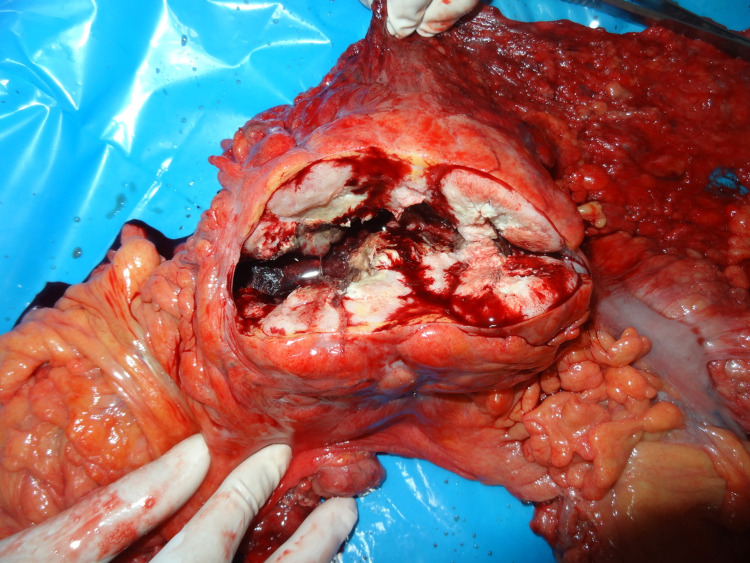
Cut section of gross showing yellowish gritty necrotic growth that is more exophytic in nature

The patient was admitted to the emergency department and on further investigation, his hemoglobin was 4 gm/dL. His serum carcinoembryonic antigen (CEA) level was 4 ng/ml, which was within the normal range (<5 ng/ml). The patient was resuscitated with six units of packed red blood cells along with fresh frozen plasma and platelets. After admission, as there was no further episode of bleeding and after achieving hemoglobin of 10.4 gm/dL, for tissue biopsy, the patient was taken for colonoscopy and also to rule out any synchronous lesions. Colonoscopy was suggestive of transverse colon mass and the scope was not further negotiable with no distal synchronous lesion. Although there was no new episode of haematochezia and the patient's vitals were stable, given the high risk of rebleeding and related complications, we decided to take the patient for urgent colectomy. For the same reason, we did not wait for the biopsy report. On exploration, we found a proximal transverse colon mass. Extended right hemicolectomy with handsewn side-to-side ileocolic anastomosis was performed (Figures 2, 3) as the patient had a good performance status, was intraoperatively stable, and had no visceral or peritoneal metastasis.

**Figure 4:**
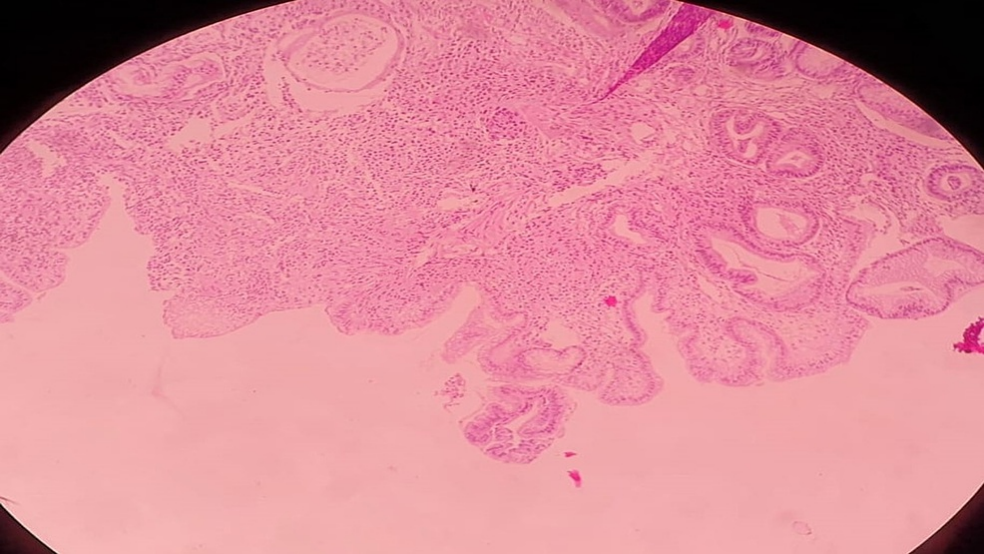
Histopathology slide showing NET invading the submucosa (hematoxylin-eosin staining) NET: neuroendocrine tumor

**Figure 5:**
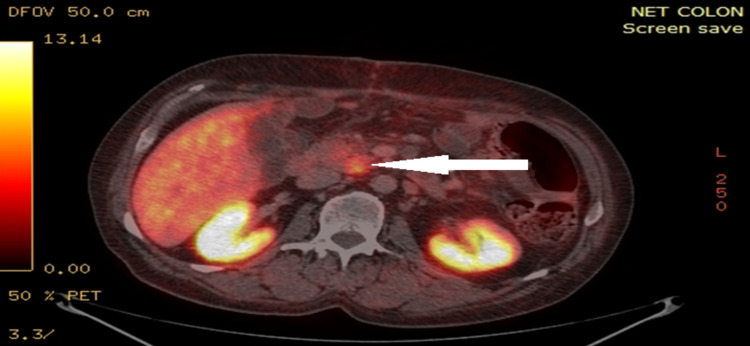
Somatostatin receptor imaging with gadolinium Ga 68–DOTATATE PET/CT at third month postoperatively The image shows high isotope uptake of retroperitoneal lymph node (arrow) suggestive of metastatic disease PET/CT: positron emission tomography/computed tomography

The postoperative period was uneventful and the patient was discharged on postoperative day eight. The histopathological report was suggestive of a high-grade NET (Figure 4) with stage pT3N1M0 (Ki-67 80-90% with 20-40 mitosis/10 HPF); 12/17 lymph nodes were positive for metastasis, synaptophysin-positive, and negative for chromogranin. Serum chromogranin and urinary 5-HIAA were normal. Platinum-based adjuvant therapy was started in the follow-up period. After three months, somatostatin receptor imaging with gadolinium Ga 68-DOTATATE PET/CT was suggestive of metastasis to retroperitoneal lymph node (Figure 5) and omentum leading to a diagnosis of disseminated disease with overall survival of the patient of only six months.

## Discussion

NETs are a rare condition with low malignant potential that arise from neuroendocrine cells, which primarily originate from neural crest cells. The most commonly involved sites are the gut, lungs, pancreas, thymus, testis, and ovaries [1]. In CRC, adenocarcinoma accounts for more than 90% of cases [7]. A large number of colonic NET cases have been detected recently, but they are still rare. Of all cases of CRCs, 0.3% are colonic and 1% are rectal NETs. Similarly, of all cases of gastrointestinal (GI) carcinoids, colonic NETs account for approximately 12% with the most common location being the cecum and the right-side colon (48%) and the second most common being rectosigmoid (43%) [7]. In our case, it was the transverse colon, which is not among the most common sites.

According to the WHO criteria, all GI NETs are categorized into three grades based on the degree of differentiation and histology: grade 1 (low grade, well-differentiated), grade 2 (intermediate grade, well-differentiated), and grade 3 (high grade, poorly differentiated) [1]. Low- and intermediate-grade tumors include carcinoid tumors or NETs that are well differentiated, whereas high-grade tumors that are poorly differentiated include NEC [1]. Colonic NETs usually manifest in the seventh decade of life [6]. Less similar to right-sided colonic adenocarcinomas, which usually present with lethargy, weight loss, and anemia, colonic NETs, due to their larger size, commonly present with abdominal fullness, weight loss, and obstructive symptoms [4]. In rare cases, they present with haematochezia as the growth pattern of these tumors are exophytic [5]. Anemia is more commonly manifested due to chronic blood loss, and in rare cases, these patients can present with superimposed acute blood loss leading to emergency presentation. In our case, the patient presented with hematochezia. Colonic NECs are aggressive in nature in comparison to NETs from other sites. Most of them are larger in size with 40% of them being metastatic at the time of presentation [8]. In our case, as it was an emergency presentation, we did not wait for the biopsy report to conduct the surgery. Within three months of surgery, it progressed to metastatic disease, which was detected on somatostatin receptor imaging with gadolinium Ga 68- DOTATATE PET/CT. Due to the condition's delayed presentation and aggressive nature, a well-established adjuvant therapy along with radical surgery is the treatment being pursued at present. Currently, there is no well-established adjuvant therapy for this disease due to the limited numbers of cases and studies in the literature [6,9]. Many studies are in progress with respect to chemotherapy and targeted therapy [10]. Studies have shown the median survival of these tumors to be around 14.5 months [6]. Our patient underwent platinum-based chemotherapy, but due to poor response to the present chemotherapy regimen and poor biology of the disease, he had an overall survival of six months only.

## Conclusions

Colonic NECs are rare entities and associated with poor disease biology; they can present with hematochezia leading to emergency presentations. Although the surgical management is similar to that of adenocarcinoma of the colon, due to its aggressive nature and rarity, the therapeutic strategy depends on early detection along with timely surgical intervention followed by well-established adjuvant therapy, which is possible with a multidisciplinary team approach. As there has been an increase in the incidence of such cases, it is the need of the hour to study and establish adjuvant therapy protocols for these notorious tumors.
